# Compact waveguide-integrated metasurface twist with filtering and phase shifting capabilities for millimeter-wave guided-wave applications

**DOI:** 10.1016/j.isci.2025.112479

**Published:** 2025-04-28

**Authors:** Amirmasood Bagheri, Zahra Rahimian Omam, Pei Xiao, Seyed Ehsan Hosseininejad, Mohsen Khalily

**Affiliations:** 1 5G Innovation Centre & 6G Innovation Centre (5GIC & 6GIC), Institute for Communication Systems (ICS), University of Surrey, Guildford, UK

**Keywords:** Physics, Optics, Wave physics

## Abstract

Recent advancements in metasurfaces have enabled precise control of wave properties such as amplitude, phase, and polarization, which are critical for multifunctional devices in modern communications. While most metasurfaces are designed for free-space use, this work integrates metasurfaces within waveguides to enable guided-wave control. We introduce a compact waveguide-integrated metasurface twist that achieves polarization rotation, bandpass filtering, and phase shifting. The metasurface comprises a 3×3 array of dog bone–shaped resonators and orthogonal gratings. Positioned between two rectangular waveguides with a 90° twist, it allows precise tuning of wave properties. A fabricated prototype operating in the 26.6–29 GHz band shows an average insertion loss of 0.6 dB and a minimum of 0.34 dB at 28 GHz. This work demonstrates a compact, high-performance solution for millimeter-wave (mm-wave) guided-wave systems with a device thickness of only $0.16\lambda_{g}$.

## Highlights

• Metasurface integration in waveguides enables multifunctional mm-wave devices
• Waveguide-integrated metasurface twist enables polarization rotation
• Enables polarization rotation, bandpass filtering, and phase shifting
• Compact design for next-generation mm-wave communication systems

## Graphical abstract

**Figure:**
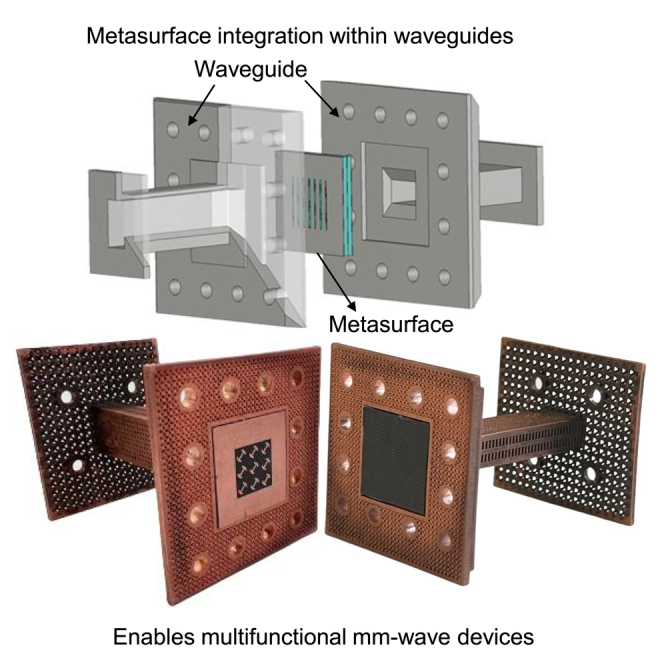

## Introduction

Recent advancements in metasurfaces have revolutionized electromagnetic wave control, enabling precise manipulation of wave properties such as amplitude, phase, and polarization.^1^^,^^2^^,^^3^ These engineered surfaces, composed of sub-wavelength unit cells, offer unprecedented control over wavefront shaping, beam steering, and filtering across a wide range of frequencies.^4^^,^^5^ Their compact size, low power consumption, and ease of fabrication have led to widespread applications in communication, sensing, and analogue computing systems, where efficient electromagnetic wave manipulation is essential.^6^ While metasurfaces are commonly used for free-space wave manipulation, their integration within waveguides remains an underexplored area, presenting an opportunity to enhance guided-wave mode control. This integration holds the potential to enable compact and multifunctional devices for advanced communication and sensing systems. Recent studies have begun to address this gap by exploring innovative metasurface designs for integrated photonics and waveguide applications, showcasing their capabilities in efficient wave manipulation, polarization control, and compact system integration.^7^^,^^8^^,^^9^^,^^10^

In microwave and millimeter-wave (mm-wave) systems, passive guided-wave components—such as waveguide twists, filters, and phase shifters—perform essential functions including polarization rotation, frequency selection, and phase adjustment.^11^^,^^12^^,^^13^^,^^14^^,^^15^^,^^16^^,^^17^ Traditionally, these components have been deployed as separate units within radar, communication, and sensing applications. However, as advanced communication technologies, including 5G and beyond, push toward higher frequencies and smaller device footprints, there is an increasing need for compact, integrated waveguide subsystems to meet these requirements.^15^^,^^18^ Recent studies have introduced single-step and multistep waveguide twists with filtering capabilities. The single-step designs achieve polarization rotation by gradually rotating multi-resonator filters, which minimizes return loss and device size.^11^^,^^12^^,^^13^^,^^19^^,^^20^^,^^21^^,^^22^^,^^23^ By contrast, the multistep designs incrementally rotate resonators to achieve broadband performance, albeit often with increased device length spanning several guided wavelengths.^14^^,^^24^^,^^25^^,^^26^ For example, in Huang et al.,^11^ a twist filter for mm-wave applications employs four rectangular resonators to achieve polarization rotation; however, its complex and bulky design—due to varying resonator heights—requires precise alignment, which limits its practical usability. Similarly, in Zhu et al.,^12^ a 3D-printed twist filter that utilizes hollow cavities improves efficiency but has a large footprint of 2.354 $\lambda_{g}$, restricting its integration into compact systems. As a result, these conventional designs are typically bulky and less suited to the growing demand for miniaturization in modern high-frequency communication systems.

**Figure 1:**
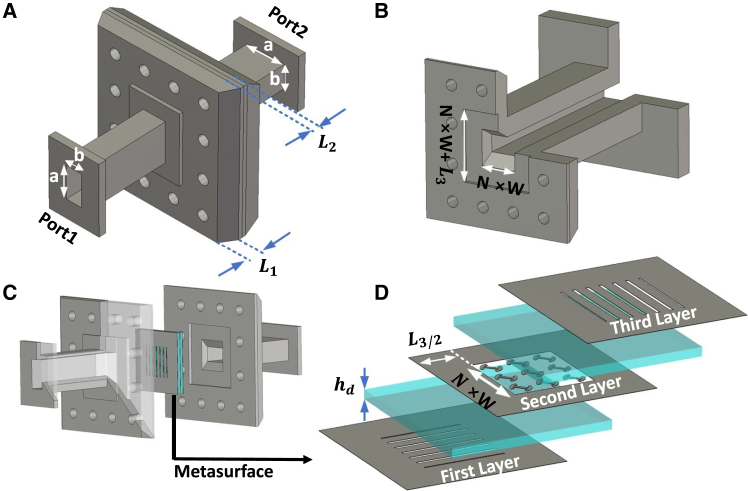
Proposed waveguide-integrated metasurface twist structure (A) 3D view illustrating the geometry. (B) Waveguide structure on the Port 2 side. (C) Extracted layout of the structure, featuring a metasurface inserted between two rectangular waveguides with a 90° rotation. (D) The metasurface sheet comprises a three-by-three array of unit cells in a multilayer design. Parameters: number of unit cells N=3, unit cell period W=3mm, $L_{1}=0.15\;\text{mm}$, $L_{2}=2.105\;\text{mm}$, $L_{3}=9\;\text{mm}$, a=8.636mm, b=4.318mm.

Building on these techniques for waveguide twists, an intriguing opportunity arises with the application of metasurfaces. In this paper, we present a novel approach that integrates metasurfaces directly within a waveguide structure, resulting in a compact, multifunctional device capable of polarization twisting, bandpass filtering, and phase shifting (Figure 1). For verification purposes, the proposed structure is designed to operate within the Ka-band frequencies, specifically from 26.6 to 29 GHz. Experimental results indicate that the integrated device achieves low insertion loss (averaging 0.6 dB) within the passband, closely aligning with simulation data. As the first waveguide-integrated metasurface twist, this approach achieves both compactness and multifunctionality through precise tuning of embedded metasurface parameters. This design not only facilitates efficient polarization rotation but also provides adjustable filtering and phase-shifting capabilities without increasing device dimensions, making it highly suitable for next-generation communication and sensing systems that demand compact, high-performance components.

The following sections of this paper detail the design, simulation, and experimental validation of our proposed structure. Results and discussion outlines the structural configuration, design process, and working principles. In this section, we describe the fabrication process and present the experimental results, showcasing the device’s performance in terms of insertion loss, polarization manipulation, and filtering capabilities, and comparing these with simulation data. Finally, conclusions are provided in Section 4.

## Results and discussion

The proposed waveguide-integrated metasurface twist is designed to perform three critical electromagnetic functions, polarization rotation, amplitude filtering, and phase shifting within a compact footprint. This section presents the step-by-step development of the device, including its structural design, working principle, and simulated performance. The effectiveness of the metasurface twist is validated through full-wave electromagnetic simulations, followed by experimental characterization of a fabricated prototype. The results confirm the successful integration of multiple functionalities in a miniaturized form, making it highly suitable for next-generation mm-wave guided-wave systems.

### Design of the waveguide-integrated metasurface twist with filtering and phase shifting capabilities

The 3D geometric configuration of the proposed metasurface-based twist integrated within a waveguide is shown in Figure 1A, with additional extracted views provided for clarity in Figures 1B–1d. This design features a compact assembly incorporating a metasurface that enables efficient wave manipulation within a waveguide environment. Targeted for Ka-band frequencies, the configuration includes two standard WR-34 rectangular waveguides (8.636 mm × 4.318 mm), positioned orthogonally, with one serving as the input port (Port 1) and the other as the output port (Port 2). The cross-section of the twist section has dimensions N×W, and the structure extends a length of $L_{3}=9\;\text{mm}$ to accommodate the twist while minimizing return loss. Parameters N and W define the number of unit cells and the unit cell period, which are N=3 and W=3mm, ensuring compatibility with the WR-34 waveguide dimensions. Figure 1D shows a detailed view of the metasurface layout within the waveguide, highlighting its precise placement and orientation. The metasurface is considered with a thickness of $L_{2}=2.105\;\text{mm}$, complemented by two steps, each of $L_{1}=0.15\;\text{mm}$, to ensure precise positioning. Note that in Figure 1, $L_{1}$ appears larger than its actual dimension for clarity. All simulations are conducted using CST Microwave Studio to model the electromagnetic performance of the proposed design. The simulations are performed on a workstation equipped with [specific CPU, e.g., Intel Xeon or AMD Ryzen processor] and [specific RAM, e.g., 64 GB RAM], with computational times of approximately 2 h, depending on the complexity of the model and frequency sweep range.

**Figure 2:**
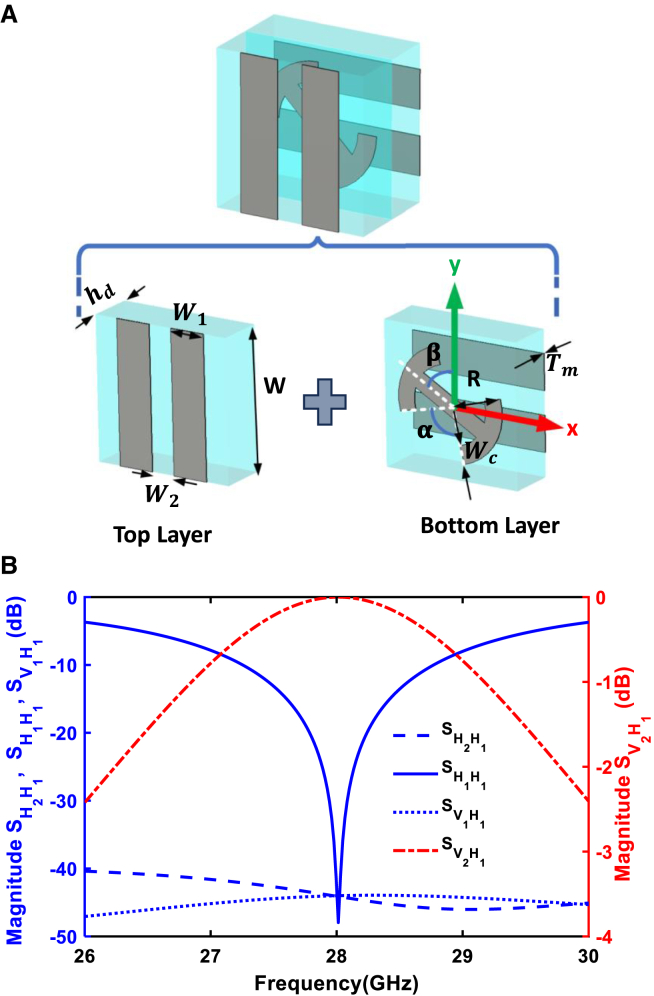
Metasurface unit cell design and polarization conversion performance (A) Unit cell of the metasurface with parameters: W=3mm, $W_{1}=1.15\;\text{mm}$, $W_{2}=0.35\;\text{mm}$, $h_{d}=1\;\text{mm}$, R=1.25mm, $W_{c}=0.35\;\text{mm}$, $T_{m}=0.035\;\text{mm}$, α=90°, and β=45°. (B) Simulated S-parameters of the metasurface unit cell (H: Horizontal, V: Vertical).

Figure 2A illustrates the unit cell of the metasurface, which integrates a dog bone–shaped resonator and two perpendicular metallic gratings. These gratings, positioned above and below the resonator, form a Fabry-Perot cavity that enhances the electromagnetic properties of the structure, particularly for polarization manipulation. The unit cell comprises three metallic layers, labeled as the first, second, and third layers, separated by two dielectric layers of Teflon glass board (F4B) with a relative permittivity of $\varepsilon_{r}=2.65$ and a loss tangent of tanδ=0.003. Each dielectric layer has a thickness of $h_{d}=1\;\text{mm}$, providing structural support for the multilayer configuration. Adjustable parameters α=90° and β=45° define the arc angle of the dog bone-shaped arm and its rotational angle relative to the y axis, respectively. The first layer of metallic strips transmits waves polarized in the x− direction while reflecting waves polarized in the y− direction, whereas the third layer exhibits the opposite behavior. The proposed metasurface achieves efficient polarization manipulation through the interaction between orthogonal gratings and the central dog bone–shaped structure, which plays a critical role in the device’s functionality. The dog bone structure is based on resonant electromagnetic interactions and carefully engineered anisotropy, enabling selective coupling with orthogonal polarization components to induce the necessary phase shifts and constructive interference for polarization conversion. Specifically, the orthogonal arms of the resonator are engineered to create differential responses for horizontal and vertical polarization components. This design facilitates polarization rotation through constructive interference of phase-shifted waves, a phenomenon critical for achieving high-efficiency polarization conversion. Additionally, the dog-bone structure enhances Fabry-Perot–like cavity effects and multireflection phenomena, amplifying the interaction between the electromagnetic wave and the metasurface. This synergy results in a highly efficient and compact polarization manipulation device, where the absence of the dog bone element would severely impair the metasurface’s efficiency and overall performance.^2^^,^^27^^,^^28^ It is important to note that the design approach is not limited to this specific pattern; any metasurface structure capable of polarization conversion and satisfying the integration and performance requirements within the waveguide can be employed. The dog bone-shaped pattern is specifically chosen for its simplicity, ease of fabrication, and effectiveness in demonstrating the proposed concept.

Figure 2B presents the simulated S-parameter results for the unit cell of the metasurface, designed for polarization conversion. The results show that there is minimal reflection in the same polarization mode, as indicated by the low values of $S_{H_{1}H_{1}}$, indicating that almost no signal is reflected back in the horizontal polarization. The transmitted signal is effectively converted to vertical polarization, demonstrated by the prominent peak in $S_{V_{2}H_{1}}$, confirming efficient polarization rotation from horizontal to vertical. Furthermore, the parameters $S_{H_{2}H_{1}}$ and $S_{V_{1}H_{1}}$ indicate the absence of transmitted horizontal and reflected vertical modes, respectively. This verifies that the unit cell effectively suppresses the original horizontal polarization in the transmission direction and minimizes any reflection in the orthogonal vertical polarization. Together, these results confirm that the metasurface structure effectively achieves polarization rotation with high transmission efficiency and minimal unwanted reflection.

**Figure 3:**
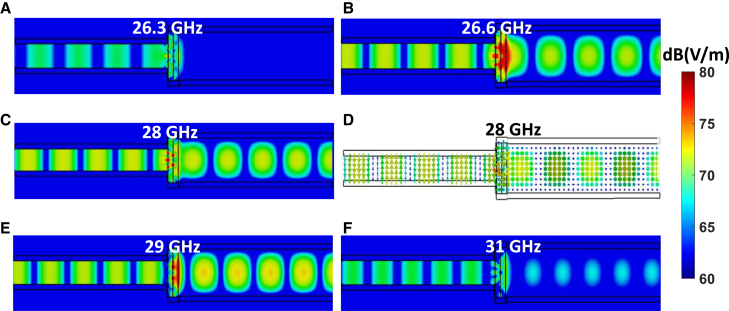
Electric field distribution of the fundamental mode $\left(TE_{10}\right)$ in the proposed structure Magnitude distributions are shown both inside and outside the operational bandwidth at (A) 26.3 GHz, (B) 26.6 GHz, (C) 28 GHz, (E) 29 GHz, and (F) 31 GHz, with the vector representation at 28 GHz depicted in (D).

The electric field distributions of the proposed structure at various frequencies, both inside and outside the operational bandwidth, are illustrated in Figure 3. Higher field intensities are observed near the waveguide’s twist interface within the operational frequency band, as demonstrated in Figures 3b, 3c, and 3e, indicating regions of strong electromagnetic interaction. In contrast, Figure 3a and 3f, which depict frequencies outside the operational bandwidth, show significantly reduced field intensities and a lack of pronounced interaction near the waveguide’s twist interface, confirming the absence of effective electromagnetic coupling at these frequencies. Figure 3d provides a vector field representation at 28 GHz, clearly showing the direction and flow of the electric field. Both the vector and magnitude representations confirm that the polarization direction of the electromagnetic wave undergoes a 90° rotation as it propagates from the input to the output port via the metasurface. This behavior highlights that the $TE_{10}$ mode is effectively guided through the twist with minimal disruption to field continuity, ensuring reliable transmission across the operational frequency band, while frequencies outside this range exhibit minimal or no interaction, supporting the design’s frequency selectivity.

**Figure 4:**
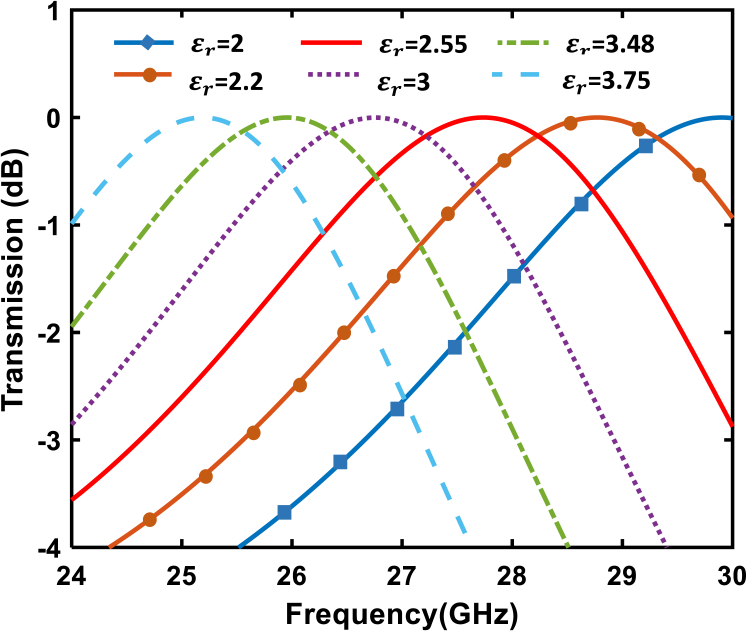
Transmission characteristics of the unit cell with different dielectric constant of the substrates, demonstrating their filtering capabilities across the 24 GHz–30 GHz frequency range

The bandpass filtering functionality of the proposed device is achieved through the resonant characteristics of the dog bone–shaped resonator and the orthogonal metallic gratings within the metasurface unit cells. The resonator geometry and material properties create a Fabry-Perot–like cavity effect, where constructive interference within the passband enhances transmission efficiency, and destructive interference outside the band limits unwanted frequencies. This principle is illustrated and supported by the simulated S-parameters shown in Figure 4, providing a clear representation of the device’s filtering performance. Building on this, Figure 4 illustrates the amplitude filtering capabilities of the proposed unit cell, depicting the transmission characteristics over a frequency range of 24 GHz–30 GHz. Each curve corresponds to a specific dielectric constant of substrates employed in the metasurface. The dielectric constants $\epsilon_{r}$ selected for the study, as presented in Figure 4, correspond to commercially available substrates to ensure practicality and ease of replication. The use of these materials ensures the relevance of the proposed design to real-world applications. These materials are listed in Table 1 for reference. The variation in transmission profiles—characterized by differences in center frequency— highlights the unit cell’s flexibility in amplitude manipulation. By fine-tuning these parameters, the filtering response can be precisely tailored to achieve desired performance criteria. This adaptability allows for the optimization of the twist to meet the requirements of specific functions such as signal filtering and frequency-dependent sensing.

**Table 1:** Selection of dielectric constants and practical substrate materials

$\epsilon_{r}$	2	2.2	2.55	3	3.48	3.75
Substrate	Rogers RO3003	Duroid 5880	Isola Astra MT77	Rogers RO3006	Rogers RO4350B	Taconic TLY-5A

**Figure 5:**
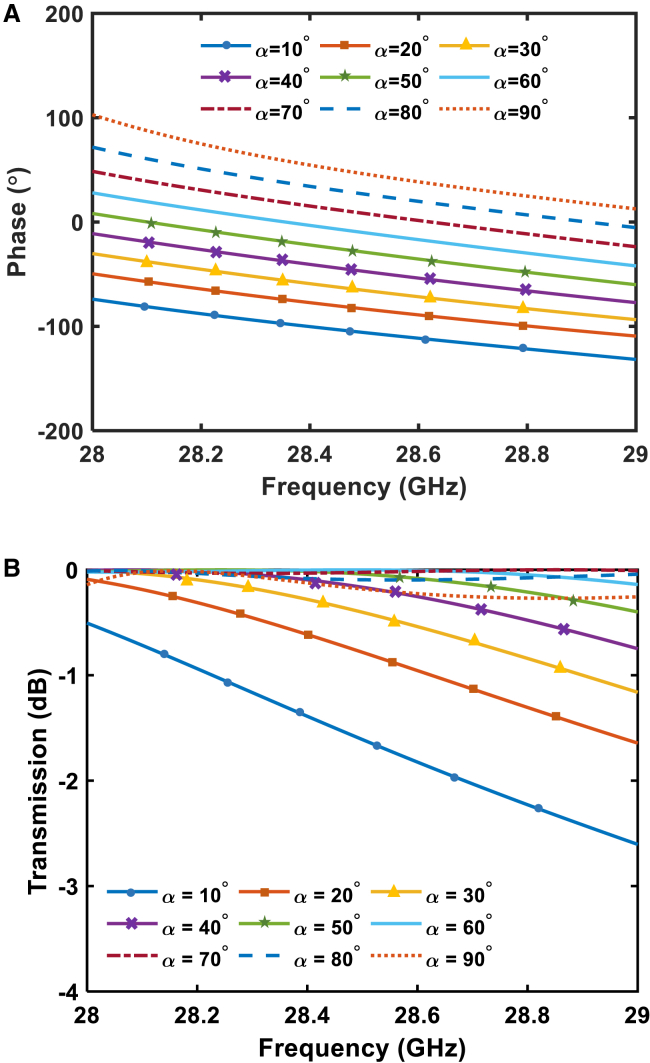
Phase and amplitude response of the metasurface under geometric tuning (A) Phase response of the metasurface by changing α (B) Transmission coefficient. (substrate is standard F4B with $\varepsilon_{r}$ = 2.65).

Moreover, the phase-shifting functionality is realized by the relative orientation of the parameter α. Such changes induce a differential phase shift as the wave propagates through the metasurface. By fine-tuning these parameters, the metasurface achieves precise phase control across the target frequency range. This phenomenon is detailed in Figure 5, which provides a clear representation of the phase response. The phase response of the twisted waveguide and the transmission coefficient as a function of the twist angle α are depicted in Figures 5A and 5B, respectively. This capability highlights the metasurface’s effectiveness in preserving signal integrity and ensuring phase coherence, even under varying operational conditions. Such features make the design highly suitable for advanced applications requiring precise phase control, amplitude filtering, and polarization rotation across the mm-wave frequency spectrum.

### Fabrication and measured results

**Figure 6:**
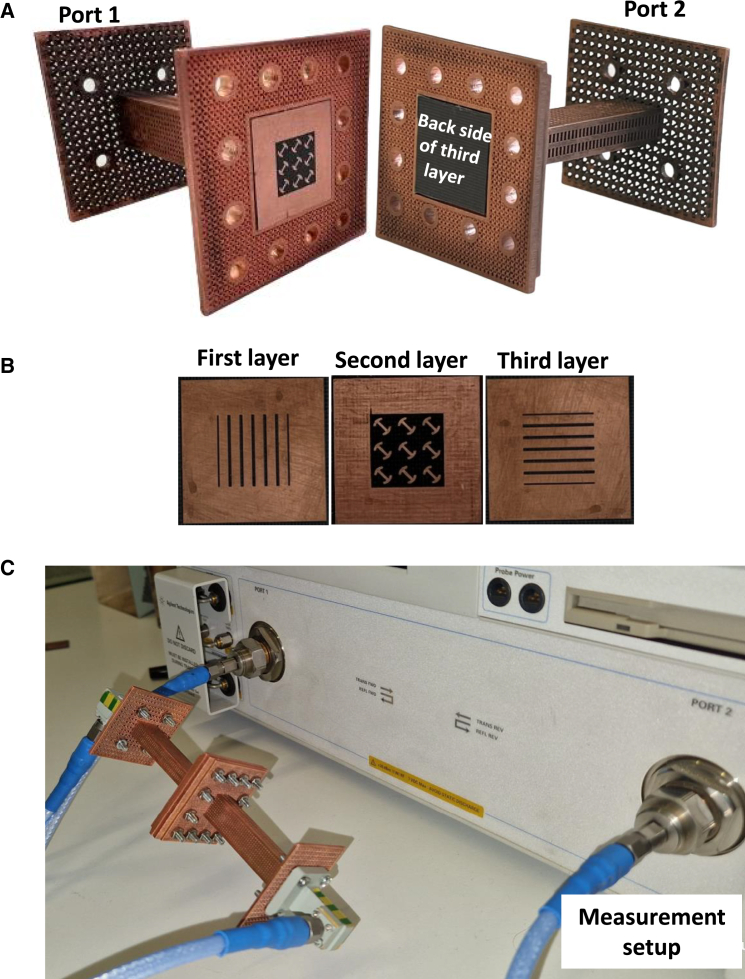
Fabricated prototype and experimental setup of the waveguide-integrated metasurface twist (A) 3D depiction of the proposed waveguide twist-filter and positioning for integrating the metasurface within the waveguide. (B) Metasurface structure consisting of three metallic layers. (C) Assembled waveguide twist-filter and its measurement setup.

Measurements are conducted at the University of Surrey, U.K., using an Agilent Technologies N5230A PNA vector network analyzer equipped with 26–30 GHz frequency extension heads. Figure 6A provides photographs of the fabricated waveguide-integrated twist, while Figure 6B illustrates the detailed metasurface structure, highlighting its three metallic layers and three-by-three unit cell multilayer design. The waveguides are fabricated using 3D printing technology and coated with copper, while the metasurface is fabricated using standard printed circuit board (PCB) technology to ensure reliability and scalability. The fabricated metasurface has a total thickness of 2.405 mm, corresponding to 0.16 $\lambda_{g}$. A key challenge during fabrication is ensuring the accurate alignment of the metasurface layers within the waveguide, as minor misalignments could significantly affect performance. Additionally, Figure 6C showcases the assembled twist filter alongside its Ka-band test setup, offering a comprehensive view of the experimental configuration.

**Figure 7:**
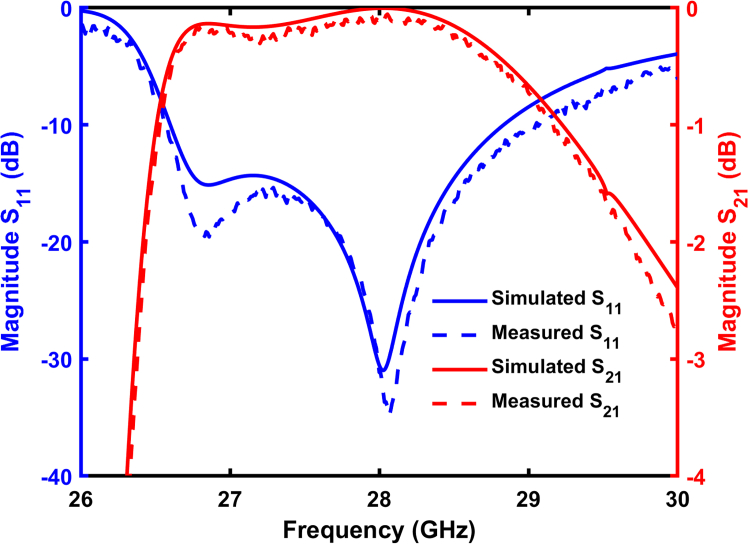
Simulated and measured S-parameters of the waveguide-integrated metasurface twist with filtering capability

Figure 7 displays the simulated and measured S-parameters of the 90°waveguide-integrated twist with bandpass filtering. The results indicate that $S_{11}<-10\;\text{dB}$ is consistently maintained across the operating frequency band (26.6–29 GHz), yielding a fractional bandwidth of 8.7%. The average insertion loss measured across the band is only 0.6 dB, with a particularly low insertion loss of 0.34 dB at 28 GHz. The measured results closely match the simulations, affirming the excellent performance of the waveguide twist-filter in the Ka-band. Minor discrepancies between simulated and measured insertion losses are attributed to surface roughness, which reduces effective conductivity, and to uncertainties in the dielectric loss parameters used in simulation. These findings confirm that utilizing an electrically thin metasurface integrated in waveguide enables the design of a compact guided-wave twist that also functions as a bandpass filter. This dual functionality is highly advantageous for applications that require both spatial and spectral control of electromagnetic waves, such as advanced communication systems and radar technologies.

Despite its promising performance, several practical challenges must be addressed to optimize the design for real-world applications. A key challenge is the power-handling capability of metasurface structures, which is often limited by substrate materials with low breakdown voltages. Using alternative materials with higher thresholds could mitigate this limitation. Another challenge involves electromagnetic leakage caused by misalignment and air gaps during assembly, which reduces efficiency. Ensuring robust integration and minimizing air gaps is critical to overcoming this issue. Furthermore, higher operating frequencies demand greater precision in PCB fabrication, as minor deviations can significantly degrade performance. Advanced fabrication techniques are therefore essential to achieve and maintain the necessary structural accuracy.

A comprehensive comparison of the characteristics and performance of the proposed waveguide twist with integrated filtering capability, relative to other designs operating within a similar frequency range, is presented in Table 2. The table evaluates the design’s functionality in terms of polarization rotation and frequency selection (including band-pass filter (BPF) or low-pass filter (LPF)). This table illustrates that our proposed structure, incorporating a meticulously engineered metasurface, accomplishes both polarization rotation and bandpass filtering in a notably compact form factor with reduced complexity compared to alternative designs. Building on this, the proposed waveguide-integrated metasurface twist demonstrates significant advantages in advanced mm-wave and sub-millimeter communication systems, offering a compact and efficient alternative to conventional polarization rotators and filters while maintaining high performance. Furthermore, these devices find extensive applications in satellite communication and radar systems, where their ability to enable efficient polarization conversion and compact feed network designs enhances overall system performance.

**Table 2:** Performance comparison of the state-of-the-art of waveguide twist with filtering capability

Reference	Structure	Passband (GHz)	Functions	Insertion Loss (dB)	Twist Length $\left(\lambda_{g}\right)$	Complexity of Design
Huang et al.^11^	Four rectangular resonators	27.2–28.8 (Ka)	Twist, BPF	0.61	$\lambda_{g}$	High
Zhu et al.^12^	Hollow cavities	75.0–92.6 (W)	Twist, LPF	0.48	2.354 $\lambda_{g}$	Moderate
Liu et al.^13^	Four distorted resonant cavities	14.7–15.3 (Ku)	Twist, BPF	0.85	$3.54\lambda_{g}$	Moderate
Zhang et al.^14^	Four rotated resonator cavities	31.4–32.4 (Ka)	Twist, BPF	0.84	$2\lambda_{g}$	High
Sun and Xu^19^	Air-gapped waveguide	–	Twist, Without Filtering	0.3	$2.68\lambda_{g}$	High
This Work	Metasurface	26.6–29 (Ka)	Twist, BPF	0.6	0.16 $\lambda_{g}$	Low

### Conclusion

In conclusion, this work presents a novel approach to integrate metasurface structures within waveguides, offering a compact and multifunctional solution for mm-wave guided-wave applications. The proposed waveguide-integrated twist, based on a metasurface that incorporates a dog bone–shaped resonator and two perpendicular metallic gratings, achieves precise polarization rotation, bandpass filtering, and phase-shifting capabilities within a compact form factor, with a metasurface thickness of only 0.16 $\lambda_{g}$. This integration of metasurfaces within waveguides addresses the challenges of traditional bulky waveguide designs, providing a pathway toward miniaturized, high-performance components suitable for advanced communication systems, such as 5G and beyond, as well as for various sensing and computing applications. The ability to adjust metasurface parameters for tailored performance further enhances the adaptability of this technology, making it a promising solution for future integrated electromagnetic devices.

### Limitations of the study

The study faces limitations in power handling due to substrate constraints, electromagnetic leakage from misalignment, and fabrication precision at high frequencies. Surface roughness affects conductivity, increasing insertion losses, while uncertainties in dielectric loss parameters introduce minor performance variations. Addressing these challenges through improved materials, fabrication, and integration techniques could enhance real-world applicability.

## Resource availability

### Lead contact

Further information and resources related to this study will be fulfilled by the lead contact Dr. Mohsen Khalily (m.khalily@surrey.ac.uk) upon reasonable request.

### Materials availability

This paper did not generate new unique reagents.

### Data and code availability

• Data reported in this paper will be shared by the lead contact upon request.
• This paper does not report original code.
• Any additional information required to reanalyze the data reported in this paper is available from the lead contact upon request.

## Acknowledgments

The work of Z.R.O. was supported by the United Kingdom’s Departement of Science, Innovation and Technology (10.13039/100031278DSIT) under SCONDA (https://uktin.net/SCONDA).

## Author contributions

A.B. designed the waveguide-integrated metasurface twist. A.B., Z.R.O., and S.E.H. conducted the analysis, measurement campaign, and drafted the manuscript, with M.K. and P.X. assisting in revisions. The project was supervised by M.K.

## Declaration of interests

The authors declare no competing financial interests.

## STAR★Methods

### Key resources table

**Table:** 

REAGENT or RESOURCE	SOURCE	IDENTIFIER
**Software and algorithms**		

CST Studio Suite	Dassault Systèmes	CST 2024
MATLAB	The MathWorks, Inc	R2024a

**Other**		

Vector Network Analyzer	Keysight Technologies	N5230A

### Method details

#### Numerical analysis

All the numerical analysis for this work was performed using CST Design Studio Suite 2024. A compact metasurface unit cell, consisting of a dog bone-shaped resonator and orthogonal metallic gratings, was designed to achieve polarization rotation and amplitude filtering near 28 GHz. A 3×3 array of these unit cells was embedded between two WR-34 waveguides with a 90° rotation to form a multifunctional twist structure. Full-wave simulations analyzed S-parameters, field distributions, and phase response to verify the polarization twisting, filtering, and phase-shifting capabilities. Parametric sweeps on rotation angle and substrate permittivity further optimized performance and guided the final design.

#### Sample fabrication

The proposed waveguide-integrated metasurface twist was fabricated using a combination of 3D printing and standard PCB manufacturing techniques. The waveguide sections were 3D printed and subsequently coated with copper to ensure good conductivity and compatibility with millimeter-wave operation. The metasurface itself consisted of three metallic layers forming a 3×3 array of unit cells, fabricated on Teflon glass (F4B) substrates using PCB technology. Each layer was precisely aligned and stacked to maintain structural integrity and functional accuracy. The complete metasurface assembly was then inserted between two WR-34 waveguides oriented at a 90° angle to realize the twist structure. Electrical connections and physical alignment were carefully handled to minimize insertion loss and leakage.

#### Experimental measurements

The experimental validation of the waveguide-integrated metasurface twist was carried out using an Agilent N5230A PNA vector network analyzer equipped with 26–30 GHz frequency extension heads. The fabricated prototype was placed between two WR-34 waveguide ports, and the S-parameters were measured to assess its performance in terms of insertion loss, return loss, and polarization rotation. The measurement setup ensured accurate alignment of the metasurface within the waveguide to minimize leakage and reflections. The response was measured across the Ka-band, and results were recorded for various frequencies, including the design frequency of 28 GHz. The measured data showed close agreement with simulation, confirming effective polarization rotation, low insertion loss (0.6 dB average), and consistent bandpass filtering characteristics, thereby validating the functionality of the integrated metasurface twist.

### Quantification and statistical analysis

There are no quantification or statistical analyses to include in this study.
